# Sexual Violence and Alcohol Intake: A Population-Based Explorative Study in a Northwestern Italian Area

**DOI:** 10.3390/medicina59122098

**Published:** 2023-11-29

**Authors:** Barbara Mognetti, Federica Di Scipio, Giuliana Abbadessa, Giulia Carnino, Antonella Canavese, Paola Castagna, Federica Romano, Sarah Gino, Giovanni N. Berta

**Affiliations:** 1 Department of Life Sciences and Systems Biology, University of Turin, Via Accademia Albertina 13, 10123 Turin, Italy; barbara.mognetti@unito.it; 2 Department of Clinical and Biological Sciences, University of Turin, Regione Gonzole 10, 10043 Orbassano (TO), Italy; federica.discipio@unito.it (F.D.S.); giuliana.abbadessa@unito.it (G.A.); 3 Corso di Laurea in Medicina e Chirurgia at Azienda Ospedaliera Universitaria San Luigi (Orbassano), University of Turin, v. Verdi 8, 10124 Turin, Italy; carninogiulia@gmail.com; 4 Centro Soccorso Violenza Sessuale, Presidio Ospedaliero Sant’Anna, Città della Salute e della Scienza, Corso Spezia 60, 10126 Turin, Italy; acanavese@cittadellasalute.to.it (A.C.); pcastagna@cittadellasalute.to.it (P.C.); 5 Department of Surgical Sciences, University of Turin, Corso Dogliotti 14, 10126 Turin, Italy; 6 Section of Periodontology, C.I.R. Dental School, Department of Surgical Sciences, University of Turin, Via Nizza 230, 10126 Turin, Italy; federica.romano@unito.it; 7 Department of Health Sciences, University of Eastern Piedmont, Via Solaroli 17, 28100 Novara, Italy; sarah.gino@uniupo.it

**Keywords:** sexual violence, rape, alcohol intake, risk factors

## Abstract

*Background and Objectives*: Sexual violence (SV) is a major global public health concern. While socioeconomic factors and familial relationships have been widely reported to contribute to SV, the role of alcohol consumption should not be ignored. Indeed, alcohol can impair cognition, distort reality, increase aggression, and ease drug-facilitated sexual assault. This retrospective study aims to explore the relationship between alcohol consumption and SV by examining the prevalence, characteristics, and consequences of violence episodes. *Materials and Methods*: A total of 1481 women accessed the Rape Centre “Centro Soccorso Violenza Sessuale” in Turin, Italy between 2008 and 2019, with 223 reporting alcohol consumption before the assault. *Results*: The alcohol group had a younger age profile, predominantly within the 18–25-year-old category. SV incidents involving alcohol consumers were more likely to occur in public places or in someone else’s home, while the non-alcohol-consuming group experienced more violence in their own homes. Acquaintances and unknown individuals were primarily responsible, whereas partners were the most common perpetrators of violence against non-alcohol-consuming women. Alcohol consumers sought medical attention sooner after the assault and exhibited more symptoms and injuries, particularly of neurological origin. Concurrent use of recreational drugs was higher among alcohol consumers. The logistic regression analysis revealed higher odds of injury for Italian women and those in the 18–35 age groups after consuming alcohol. *Conclusions*: This study contributes to the understanding of the relationship between alcohol consumption and SV. The prevalence of alcohol-related sexual aggression is lower compared to that shown in previous studies. Nationality, age, and assailant identity influence SV dynamics. These findings can guide well-targeted interventions and prevention strategies to address SV and inform communities facing similar challenges.

## 1. Introduction

Sexual acts that are not consensual and are frequently acquired through force, coercion, or the use of psychological and/or physical intimidation can happen to individuals of all ages and genders and are classified as sexual violence (SV). SV is a severe global public health problem with significant physical, psychological, and social consequences for survivors. While it can happen to anyone, women are disproportionately affected with up to one-third of them experiencing sexual violence at some time in their lives [1]. In 2019, the Centre for Disease Control and Prevention (CDC) identified different risk factors for SV; among them, socioeconomic status, familial relationships, and racial and ethnic background are of paramount importance [2]. However, as emphasised in previous studies [3,4,5,6,7,8], the role of alcohol consumption should not be neglected, since many sexual assaults involve alcohol intake by the victim, the perpetrator, or, more commonly, both [9].

The relationship between alcohol consumption and SV is complex and multifaceted, with various factors related both to the effects of alcohol and to its social and cultural significance that may contribute to the likelihood and nature of SV [10,11]. Alcohol effects include a reduction in cognitive and physical functioning that, in turn, impairs self-control, with the consequent effect of reducing the ability to solve conflicts non-violently [12]. Moreover, it induces a misperception of reality, reduces the perceptive capacity regarding dangerous situations and leads to difficulty in opposing resistance [13]. Furthermore, in some people, excessive alcohol consumption increases sexual desire and emotional instability [14,15]. In particular, it has been observed that individuals of the male gender, as a result of ethanol intake, tend to have aggressive attitudes, resulting in the perpetration of sexual impositions [11,16]. The mediation of alcohol and psychoactive substances in sexual interactions is a widespread phenomenon. Drug-facilitated sexual assault (DFSA) is a form of sexual violence in which drugs or alcohol are used to incapacitate the victims, making them more vulnerable to assault. DFSA is a particularly insidious form of sexual violence, as it can be difficult for victims to remember or accurately report what has happened to them [17]. In recent years, there has been growing concern about the use of drugs such as Rohypnol or Gamma Hydroxy Butyrate (GHB) in DFSA, as well as the potential for other drugs, such as benzodiazepines or opioids, to be used in this context [18]. Alcohol is often consumed in combination with cannabinoids or benzodiazepines for recreational purposes [19,20,21,22]. The effects of these substances can impair judgement, decrease inhibition, and cause memory lapses, leaving individuals vulnerable to sexual coercion or assault. Some psychiatric medications, such as antidepressants and antipsychotics, can affect reactivity and alter cognitive and emotional functioning, potentially increasing the risk of sexual assault [23,24]. Moreover, women with mental health disorders may face additional barriers to protecting themselves from SV, including social isolation, stigma, and the lack of a support system. It should be also taken into consideration that, as demonstrated in our previous work that, sometimes, women regularly consume drugs that act on the central nervous system (CNS) for therapeutic purposes, and the synergistic effects of these drugs can amplify the inhibitory effects of alcohol [25].

Despite the well-established link between alcohol consumption and SV, few studies have specifically compared the prevalence and nature of SV in women who consume alcohol compared with those who do not. This is a relevant gap in the literature, as understanding the unique risks and protective factors associated with alcohol consumption may help to inform more targeted interventions and prevention strategies, ultimately reducing the burden of this devastating public health problem.

Therefore, this observational study aims to address this gap by providing evidence on the status, characteristics, consequences, and risk indicators of SV in relation to alcohol consumption. In our previous study [25], we explored the characteristics of episodes of sexual violence in a population attending the Rape Centre “Centro Soccorso Violenza Sessuale” (SVS) at Sant’Anna Hospital in Turin, where healthcare personnel suspected the use of alcohol or drug abuse. To deepen the understanding of victimisation in sexually assaulted women who—voluntarily or involuntarily—had consumed alcohol, we expanded our previous database and introduced more stringent inclusion criteria. For this reason, we selected a population of women who had self-reported alcohol consumption, and we compared them with a non-alcohol-consuming control population.

This analysis could help to better understand a problem resulting from the intersection of global influencing factors, obtaining conclusions that are transferable to other communities sharing the same behavioural model.

## 2. Materials and Methods

### 2.1. Study Design

We conducted a retrospective cross-sectional study, based on data collected from the medical records of sexually abused female patients who were examined at the Rape Centre “Centro Soccorso Violenza Sessuale” (SVS) at Sant’Anna Hospital in Turin, Italy between 1 January 2008, and 31 December 2019. The SVS is one of the two Italian centres dedicated to violence against women (sexual violence, violence in pregnancy, violence against migrant women). In particular, it is the reference health service for the Piedmont region and is equipped with a dedicated and all-female multidisciplinary team (gynaecologists, midwives, social workers and psychologists). All women accessing the SVS at the time of the clinical/forensic evaluation for SV are routinely requested to sign written informed consent for clinical/gynaecological procedures and the collection of forensic specimens.

The research protocol complied with the ethical principles of the World Medical Association (Declaration of Helsinki) for experiments involving humans (2013) and with the General Data Protection Regulation (2018) and Provision no. 146/2019 of the Italian Privacy Guarantor. The study was approved by the Ethics Committee of the “A.O.U. Città della Salute e della Scienza di Torino—A.O. Ordine Mauriziano di Torino” (CE 112/2020) on 6 July 2020.

We consecutively enrolled all women aged 14 years or older into the study. They were allocated to the “alcohol” group if they reported consuming alcohol prior to the sexual assault, and therefore the information was available in their medical records. To select the control group, we included 320 women who reported sexual abuse and declared no alcohol intake. The control population was randomly chosen from women who did not report alcohol consumption while adhering to the distribution of the reference group over the years. Random selection was conducted to avoid introducing bias in the composition of the population concerning factors such as the average age and origin. The population represents 25% of all women who accessed the SVS for sexual violence without alcohol intake.

### 2.2. Data Collection

For issues related to confidentiality, the medical records are on paper and are kept in locked files in dedicated places accessible only by the staff of the centre. The data were extracted from one author and checked by a second author who was on the staff of the SVS Centre. Women were assigned to the “alcohol” group if they reported alcohol consumption before their sexual assault. For the control group (320 women reporting abuse but no alcohol intake), we meticulously aligned the selection with the reference group’s yearly distribution. Controls were randomly chosen, ensuring consistency with the reference group across years to prevent bias in factors like the average age and origin. Sociodemographic data and relevant information about violence episodes were gathered from the women’s health records and then registered into a database administered by the SVS staff. Considering age and nationality, women were classified into six age groups (14–17; 18–25; 26–35; 36–45; 46–55; and over 56 years of age) and categorised as “Italian” or “foreign”. The following characteristics of the episode of violence were noted: the location, the time elapsed from the violent episode to the medical examination, the relationship with the aggressor, and the number of attackers; if the woman was not able to provide information about the assailant, it was defined as “not known” (or, if more than one, “unknown group”). Data on recreational substances or drugs active on the central nervous system (CNS) that the women declared use of, occasionally or as prescriptions, were also collected.

Signs and symptoms, the types of injuries, and the body areas involved were used to classify the episode. Moreover, among the psychological conditions and psychic symptoms itemised in a checklist contained in the violence kit and registered on the medical record by specialised medical staff (psychologist or psychiatrist, as needed), we considered only those most frequently associated with the intake of alcohol (i.e., anterograde amnesia, hallucinations, numbness, confusion). The information about the type and quantity of alcohol assumed and the time between alcohol consumption and sexual violence was too limited and unreliable to be included in the study.

### 2.3. Statistical Analysis

Values of quantitative variables were expressed as the mean ± standard deviation, while values of categorical variables were presented as absolute and relative frequencies. A comparison between aggressed women who consumed alcohol or did not was conducted with the χ^2^ test for the categorical variables and by the independent *t*-test for the quantitative variables.

A multiple logistic regression analysis was designed to identify risk indicators of sexual aggression related to alcohol consumption (yes vs. no), and the location of sexual violence (own private home vs. other), which were considered outcome variables. Model building was carried out using a purposeful selection of variables strategy. Initially, a preliminary analysis was carried out using univariable models, and all variables presenting *p* values < 0.25 were entered in the multivariable models. A backward select procedure with a *p* value cut-off at 0.05 was used to identify the set of independent variables in the final models. Using this approach, risk indicators for sexual aggression mediated by alcohol consumption included nationality (dichotomised as Italian and foreign) and the age of the victim (categorised into four groups: 14–17 years, 18–25 years, 26–35 years, and >35 years), location of aggression (public place, own home, someone’s home, other), and relationship with the assailant (partner, acquaintance, unknown to the victim). Risk indicators for the location of sexual violence were nationality and age of the victim, relationship with the assailant, and alcohol intake (as dichotomous). Data were presented as the adjusted odds ratio (OR) and 95% confidence intervals (CI). Wald tests were used to estimate the statistical significance.

The data analysis was performed using SPSS version 28 (Chicago, IL, USA). *p* < 0.05 was considered statistically significant.

## 3. Results

### 3.1. Population Enrolled

**Figure 1:**
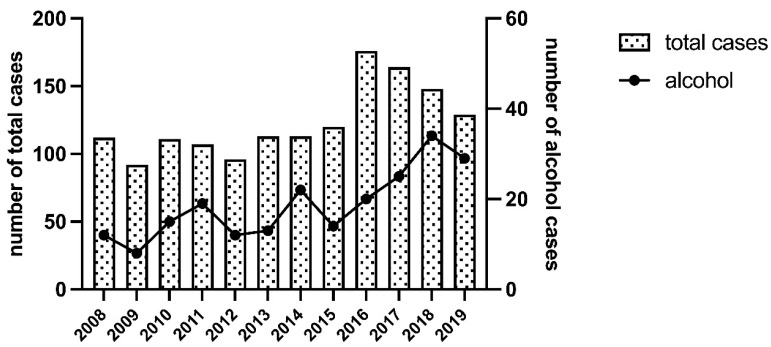
The columns refer to the left y-axis, and the black line refers to the right y-axis. The two y-axes have distinct scales to accommodate the different ranges of values for the columns and the black line.

From 1 January 2008 to 31 December 2019, a total of 1481 women accessed the SVS. Figure 1 shows the total percentage of cases of sexual aggression stratified by year and alcohol consumption.

From 2008 to 2015, the number of aggressions per year was quite constant (mean 108 ± 9.4). In 2016, the number of events peaked at 176 and was followed by a slow decline (129 events in 2019).

Two hundred and twenty-three women out of 1481 declared alcohol consumption before being assaulted. Although, between 2008 and 2019, the percentage fluctuated between 8.7% and 23%, in the last 2 years, the value was stable at over 20%. While the number of total cases decreased from 2016 to 2019, in this time frame, the number of events related to alcohol consumption increased.

**Figure 2:**
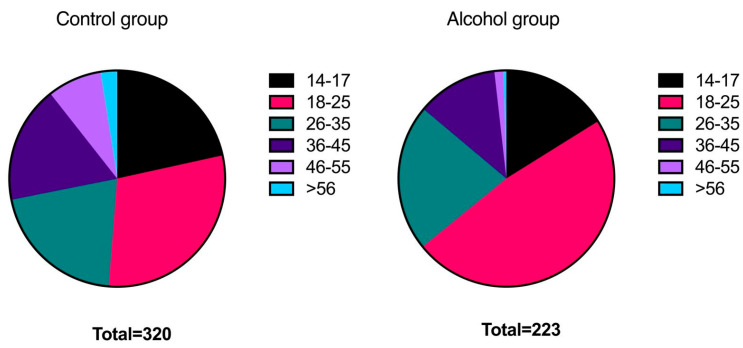
Distribution of ages in the control group (**left**) and the alcohol group (**right**).

The alcohol group (n = 223) had a mean age of 24.8 ± 8.7 years, while the control group (n = 320) had a mean age of 28.5 ± 12.1 years (*p* < 0.05). As summarised in Figure 2, the 18–25-year cohort was the most represented in both groups, although its frequency was significantly higher in the alcohol group (47.98%) compared to the control group (29.69%) (χ^2^ = 18.053, *p* < 0.001).

Similarly, there was a statistically significant difference in the percentage of women in the 46–55-year cohort, with only 1.35% in the alcohol group compared to 8.13% in the control group (χ^2^ = 10.645, *p* = 0.001).

Two hundred and nine women declared the voluntary intake of alcohol, while 14 victims were either pressured to drink or did not realise that they were drinking alcohol. Despite the small number of reported incidents, involuntary alcohol consumption was more common in women aged over 25 years old compared to the younger ones (5 vs. 9; χ^2^ = 4.007, *p* = 0.045). Spirits were consumed by 28.7% of the women, and both wine and beer were consumed by 11.7% of them. Nearly one woman out of four (24.2%) declared that she had mixed different kinds of alcoholic beverages, for the remaining 23.8% of the enrolled population, no information on the type of alcohol consumed was available.

Among women aged under 25, spirits were the primary choice of alcoholic beverage (χ^2^ = 5.445, *p* = 0.02). Moreover, the consumption of recreational substances was more prevalent among alcohol consumers in comparison to the corresponding age group in the control population, as indicated by a significantly higher rate of consumption (χ^2^ = 15.358, *p* < 0.001). Women aged over 25 who consumed alcohol reported symptoms more frequently (χ^2^ = 11.789, *p* < 0.001), and regardless of alcohol consumption, they exhibited more injuries compared to the younger ones (alcohol group: χ^2^ = 5.128, *p* = 0.024; control group: χ^2^ = 6.162, *p* = 0.013).

### 3.2. Nationality of the Enrolled Population

**Figure 3:**
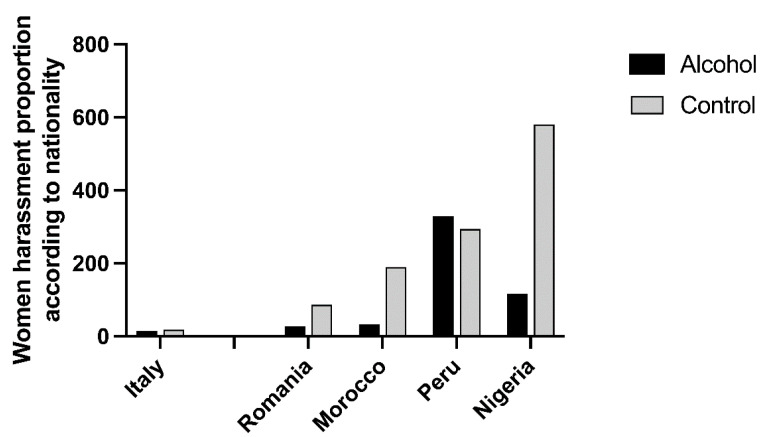
For each nationality considered, the number of women who were assaulted (with or without alcohol consumption) is expressed in relation to the average population of that same nationality who lived in the province of Turin from 2008 to 2019.

The alcohol group consisted of 146 Italian and 77 foreign women, whereas the control group was composed of 176 Italian and 144 foreign women (χ^2^ = 5.544, *p* < 0.019). Among the foreign women, those in the alcohol group originated from Europe (n = 25), America (n = 38), Africa (n = 9), and the eastern part of the world (Afghanistan, Australia, Bangladesh, China, Moldova, and Russia) (n = 5). The control group included foreign women from Europe (n = 54), America (n = 32), Africa (n = 48), and the eastern part of the world (n = 10). African women were more numerous in the control group (χ^2^ = 15.668, *p* < 0.001). Among the foreign women, the most represented nationalities were Romanian (alcohol n = 14, control = 44), Moroccan (alcohol n = 19, control = 17), Peruvian (alcohol n = 4, control = 23), and Nigerian (alcohol n = 3, control = 15). The average numbers of women from these nationalities in the province of Turin during the study period (2008–2019) were as follows: Romanian = 50,843.3; Morocco = 12,086.5; Peru = 5778.2; and Nigeria = 2587.9. The mean number of Italian women in the same period and territory was 973,133 [26]. Figure 3 displays the ratio between the number of assaulted women, proportionally referring to the numbers of women of the same nationality.

Regarding the age distribution, while the alcohol group showed an equal distribution of adults and minors among both Italians and foreigners, in the control group, minors were predominantly Italian (χ^2^ = 4.255, *p* = 0.039). Among the minors, Italian females were predominantly subjected to rape if they did not consume alcohol (χ^2^ = 3.86, *p* = 0.049), whereas no differences were observed in the foreign group. In the most represented age group, 18 to 25 years old, the number of Italian women in the alcohol group (n = 73) was significantly higher than that of foreigners (n = 34, χ^2^ = 13.96, *p* < 0.001).

African women were significantly more greatly represented in the control group (n = 48) compared to the alcohol group (n = 9; χ^2^ = 11.176, *p* < 0.001 vs. the other foreigners, χ^2^ = 15.668, *p* < 0.001 vs. all the other women, Italian included).

Italian women more frequently consumed CNS drugs when considering both the entire cohort (alcohol plus control group, 70 Italians vs. 16 foreigners, χ^2^ = 19.286, *p* < 0.001) and the control group (41 vs. 7; χ^2^ = 19.877, *p* < 0.001).

### 3.3. Location Where Sexual Violence Occurred

**Figure 4:**
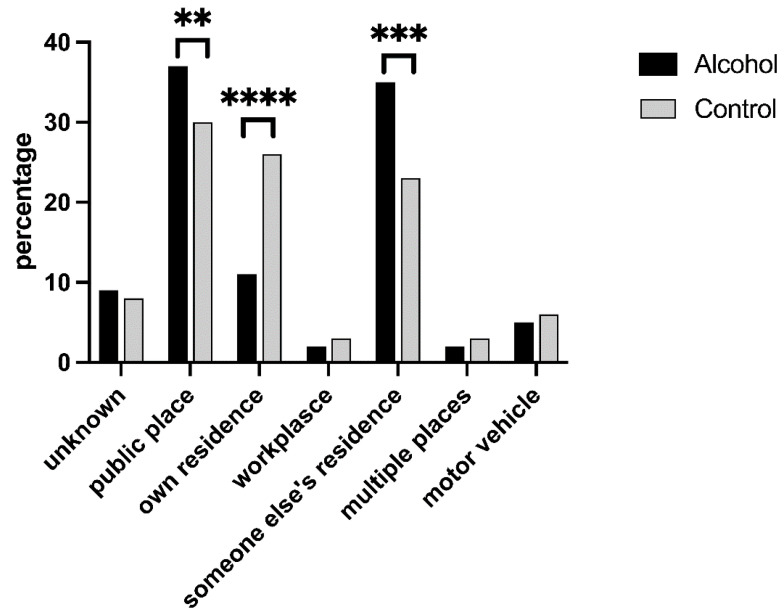
Distribution of sexual violence incidents by location. ** *p* = 0.034; *** *p* = 0.005; **** *p* = 0.001.

Among the three least frequent locations where SV was perpetrated (workplace, multiple places, and vehicles), no significant differences were found between the alcohol and control groups (Figure 4).

Conversely, in public places (36.77% alcohol vs. 30% control) and someone else’s home (34.53% alcohol vs. 23.13% control), violence was more frequently inflicted upon women who consumed alcohol (χ^2^ = 4.490, *p* = 0.034 and χ^2^ = 7.955, *p* = 0.005, respectively). Events occurring in one’s own home were significantly more common in the control group (10.76% alcohol vs. 26.25% control, χ^2^ = 18.824, *p* < 0.001).

Among women raped in a private home, those who did not consume alcohol were primarily assaulted by their partners (χ^2^ = 11.8, *p* < 0.001), while those who consumed alcohol were predominantly aggressed by acquaintances (χ^2^ = 14.248, *p* < 0.001). Similarly, when violence occurred in someone else’s home, non-drinkers were most commonly assaulted by their partners (χ^2^ = 6.072, *p* < 0.001), whereas those who consumed alcohol were more frequently targeted by unknown men (χ^2^ = 16.267, *p* < 0.001). No differences were observed between the alcohol and control groups regarding the identity of the assailant when violence was perpetrated in a public place. In cases of domestic violence, women who were raped following alcohol consumption were predominantly Italian, whereas foreign women were more prevalent in the control group (χ^2^ = 4.596, *p* = 0.032).

When considering age groups, minors and women aged between 26 and 35 years old who consumed alcohol were more frequently assaulted in someone else’s home (χ^2^ = 11.311, *p* < 0.001 and χ^2^ = 4.291, *p* = 0.038, respectively). In the 18–25-year age group, women who consumed alcohol were more frequently victimised in public places (χ^2^ = 8013, *p* = 0.004). Conversely, women who did not consume alcohol were more frequently raped in their own homes (χ^2^ = 20.705, *p* < 0.001).

### 3.4. Assailant

**Figure 5:**
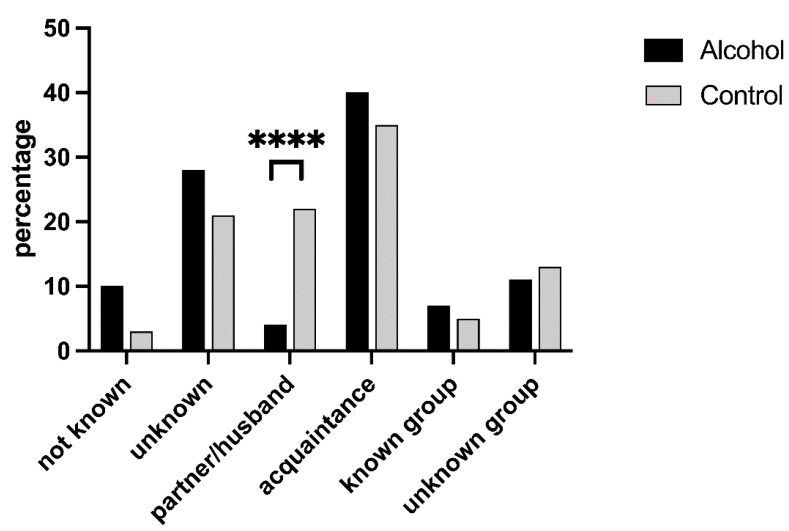
Distribution of assailant types in sexual violence incidents. The category “not known” indicates the absence of information on the assailant on the clinical report, while unknown/unknown group identifies an assailant/a group of assailants that the victim did not know. **** *p* = 0.001.

The most common perpetrators of violence were acquaintances (202 total cases) and unknown individuals (129 total cases), equally distributed between alcohol and control groups. In contrast, partners were more frequently responsible for violence against women who did not consume alcohol (4.48% alcohol vs. 22.19% control, χ^2^ = 31.071, *p* < 0.001; Figure 5). In cases involving minors, the assailant was most commonly an acquaintance, particularly if the minor had consumed alcohol (26 out of 33 cases, χ^2^ = 6.796, *p* = 0.009).

### 3.5. Time Elapsed between Rape and Medical Examination

Among the time slots examined, the period with the highest number of women seeking medical examinations was within 0–6 h following the events. This time frame was equally favoured among both the alcohol and control groups, representing a total of 126 out of 543 women.

The majority of women who consumed alcohol (62.16%, χ^2^ = 6.885, *p* = 0.009) accessed the SVS within the first 24 h after the event. In contrast, women in the control group accessed the SVS either within (50.3%) or beyond (49.7%) 24 h from the assault.

When the perpetrator was unknown, there was no difference in the timing of accessing SVS between women who consumed alcohol and those who did not. However, if the perpetrator was an acquaintance, women who had been drinking tended to access the SVS within 24 h from the event (χ^2^ = 6.294, *p* = 0.012). Italian women in the control group accessed SVS later than those who had been drinking (χ^2^ = 5.792, *p* = 0.016), while no such difference was observed among foreign women. Among the minors who consumed alcohol, 22 out of 33 accessed SVS within 24 h, which was earlier compared to those who did not consume alcohol (χ^2^ = 8.005, *p* = 0.005).

Almost all (13 out of 14) women who reported forced or unconscious alcohol consumption accessed SVS within 24 h, while this was not the case among those who voluntarily consumed alcohol (χ^2^ = 4.674, *p* = 0.031 vs. voluntary consumption).

The population that presented earlier at SVS predominantly consisted of women exhibiting symptoms (χ^2^ = 20.678, *p* < 0.001) or injuries (χ^2^ = 14.149, *p* < 0.001) without prior alcohol consumption.

### 3.6. Consumption of Drugs Active on the CNS

Table 1 presents the numbers of drugs, categorised into main groups, used by women in the alcohol and control groups. The percentage of women using CNS-active drugs did not differ significantly between the alcohol (n = 36; 15.7%) and control groups (n = 47; 14.7%), but those in the alcohol group consumed recreational drugs more frequently (χ^2^ = 5.934, *p* = 0.015) and reported lesions (χ^2^ = 4.094, *p* = 0.043). In the alcohol group, women showed also a higher consumption of benzodiazepines and Z-drugs (χ^2^ = 5.78, *p* = 0.016) as well as antidepressants (χ^2^ = 7.07, *p* = 0.008) and reported more frequent neurological symptoms (χ^2^ = 12.519, *p* < 0.001).

### 3.7. Concurrent Intake of Recreational Drugs

Fifty-four women who experienced violence after consuming alcohol (24.2% of the total) declared the concurrent intake of recreational drugs, either voluntarily (n = 36) or not (n = 18). In the control group, on the other hand, only 34 women (15.2% of the total, χ^2^ = 18.107, *p* < 0.001) declared the intake of such substances, 12 of them voluntarily and 22 involuntarily (χ^2^ = 7.065, *p* = 0.008 vs. the corresponding categories in the alcohol group).

### 3.8. Clinical Examination

**Figure 6:**
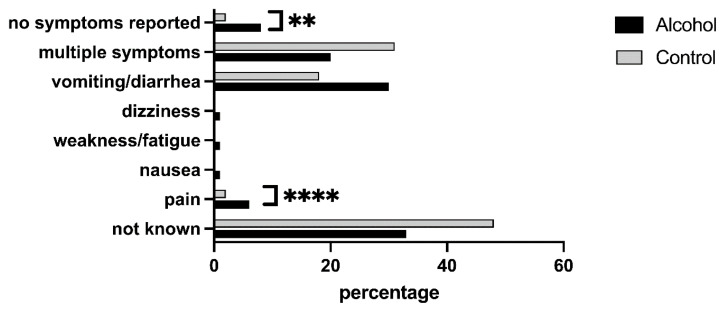
Symptoms reported during the medical examination at the SVS. ** *p* = 0.029; **** *p* = 0.001.

One hundred and sixty women in the alcohol group and 217 women (χ^2^ = 4.477, *p* = 0.029) in the control group reported symptoms when they accessed SVS (Figure 6).

The most common symptom was pain, reported by 73 women who had consumed alcohol and 154 control women (χ^2^ = 12.169, *p* < 0.001).

At the clinical examination, lesions were identified in 131 women who had consumed alcohol (62.1%) and 166 control women (52%, χ^2^ = 4.805, *p* = 0.028). The most common clinical features were multiple injuries, excluding those in the genital area, as these were reported by 53 (25.1%) women in the alcohol group and 55 (17.2%) in the control group (χ^2^ = 4.383, *p* = 0.036). No significant differences were observed between the two groups when considering the other body areas injured (head and neck, trunk, upper or lower limbs, genitals and erogenous zones/breasts, different locations including genitalia). Neurological symptoms (namely amnesia and confusion) were declared at the time of accessing SVS by 173 women who were assaulted after consuming alcohol and by 73 women in the control group (χ^2^ = 133.176, *p* < 0.001).

### 3.9. Multiple Logistic Regression Models

Adjusted assessments of factors related to sexual aggression with or without alcohol intake and the place of sexual violence are reported in Table 2 and Table 3. The multiple regression analysis showed that women with Italian nationality (OR = 1.72) and in both the 18–25 years (OR = 2.80) and 26–35 years (OR = 2.03) age groups had significantly higher odds of being injured after having drunk alcohol compared to foreign and older women (Table 2). Violence perpetrated by an unknown aggressor (OR = 5.25) or by an acquaintance (OR = 4.51) or in someone else’s private home (OR = 2.15) was more likely to occur against women who had assumed alcohol.

As reported in Table 3, there was a significantly higher chance of being sexually assaulted in one’s own home for foreign women (OR = 1.75) in the >35-year age group (OR = 3.16), aggressed by the partner (OR = 12.76), or by an acquaintance (OR = 2.80). In addition, women who did not consume alcohol were 1.72 times more likely to be aggressed in their own private home, but the association was close to statistical significance.

## 4. Discussion

We conducted a retrospective evaluation of rape events involving women who accessed the SVS centre after consuming alcohol before the assault. Far from blaming the victims who have consumed alcohol, the present study aims to provide a more in-depth analysis of the profile and context of these episodes of violence and to identify if such an assault has similar or different features compared to an analogous episode perpetrated in the absence of alcohol. This is a relevant issue since women have increased their alcohol consumption, which was, until the recent past, an exclusive feature of males. In the United States, between 2001 and 2013, there was a 16% increase in the proportion of women drinking alcohol, a 58% increase in women with heavy alcohol consumption (versus 16% for men), and an 84% increase in the annual prevalence of alcohol use disorders in women (compared to 35% in men) [27]. According to the periodical survey performed by the Italian official statistical institute [28], in 2020, in Italy, 56.2% of women over the age of 11 had consumed at least one alcoholic beverage during the year. Ethanol consumption is often adopted as a form of emancipation and a manifestation of adequacy of their role in society [29]. However, women are characterised by a greater vulnerability to the effects of ethanol due to the lesser extent of the gastric isoform of alcohol dehydrogenase [30]. The short-term effects of this substance mainly involve the CNS. Firstly, it causes an excitatory action on the brain functions, which, as the concentration of ethanol in the blood increases, becomes depressant through the interaction with the neurotransmitter systems that regulate the mechanism of cortical inhibitory control. GABAergic (inhibitory) transmission is potentiated by ethanol through the facilitation of binding between GABA and its receptor, also at small concentrations (5–50 mM) [31].

We analysed the number of events referred to the SVS at Sant’Anna Hospital in Turin. The total number of women accessing the SVS was nearly constant in the years before 2016, when a peak occurred. While the number of the total cases decreased from 2016 to 2019, in this time frame, the number of events related to alcohol consumption increased. This trend partially overlaps with what is reported in Li’s [32] meta-analysis, where the rate of sexual violence against women was found to be significantly increased from 2010 to 2019 (0.33, 95% CI = 0.27–0.37) compared to in the period from 2001 to 2009 (0.20, 95% CI = 0.16–0.25). The frequencies reported in our study are comparable, during the overlapping period, to those reported by Ricard-Gauthier [33] at the Emergency Obstetrics and Gynaecological Unit of the Geneva University Hospitals. However, the population size of Geneva in that period was less than 200,000 (UN data), while the city of Turin, where the SVS is located, had a population that was four times larger (ISTAT data). From 2010 to 2017, a total of 2065 cases of sexual assault were documented [34] at the Centre for Victims of Sexual Assault at the Edith Wolfson Medical Centre, which caters to a significant population from the Tel-Aviv area and its neighbouring suburbs in Israel. It is noteworthy that the population of Tel-Aviv during the study period was approximately 430,000 individuals (UN data). In the study conducted by Morgan and Long [35], it is documented that between 1 July 2011 and 30 June 2016, a total of 7082 individuals presented to three sexual assault referral centres in Greater London following alleged sexual assaults. However, it is challenging to directly compare these data with ours, as the study does not provide stratification of the victims based on sex. A substantial increase in hospital visits related to sexual violence was also reported in a cross-sectional study [36] utilising sexual assault data from the US Nationwide Emergency Department spanning from 2006 to 2019. The study revealed a remarkable rise of over 1533.0% in sexual-assault-related emergency department visits, with numbers escalating from 3607 cases in 2006 to 55,296 cases in 2019. Therefore, considering the aforementioned studies, despite the evident differences among the contexts examined, we observed a lower proportion of women seeking assistance at the rape centre in Turin.

Throughout the 12 years of the present survey, about 15% of the women entering the SVS declared alcohol consumption before being assaulted, and most of them declared voluntary intake. This percentage is lower compared with previous data in the literature. Ricard-Gauthier [33] found that 48% of sexually assaulted patients reported alcohol consumption. Abbey’s study reported that approximately half of all sexual assault victims declared that they were drinking alcohol at the time of the assault, with estimates ranging from 30 to 79 percent [37]. It is largely known that alcohol promotes the likelihood of impulsive/inappropriate action by suppressing the ability to anticipate and to consider the negative immediate and delayed consequences of such actions [38]. Moreover, alcohol-induced cognitive impairments might reduce one’s ability to evaluate risk and its motor weakening effects might limit the ability to resist effectively. However, in our study, a causal link between alcohol and vulnerability to violence was not established.

Numerous studies [39,40,41] have established a strong association between sexual violence perpetrators and their victims who are under the influence of alcohol or other drugs, regardless of the perpetrators’ own substance use. Consequently, individuals who have experienced sexual assault may be coerced into consuming alcohol or unknowingly subjected to its administration. In such instances, the term drug-facilitated sexual assault (DFSA) is employed to describe these occurrences. In our study, the percentage of individuals who reported forced or unaware consumption of alcohol is 6.3%. Nevertheless, with the present data, we cannot state if, in our population, alcohol is a favouring factor.

In the current study, the mean age of the population who declared alcohol consumption is lower than that of the control population. These findings align with the concerns raised by the global health organisation highlighting the increasing trend of alcohol consumption among youth and the progressively younger age of alcohol consumers [42,43]. It deserves to be underlined that the legal drinking age in Italy is 18, which means these young women were illegally consuming alcohol.

The alcohol group comprised nearly half of the women aged 18 to 25, while this age range represents one-third of the control group. It could be argued that the most represented age cohort in the alcohol group corresponds to the age range when nightclubbing is more frequent, often involving alcohol consumption. Moreover, the control group consisted equally of Italian and foreign women, whereas in the alcohol group, Italian women constituted two-thirds of the population. It could be hypothesised that foreign women, due to cultural or religious reasons (11% of our sample consists of women from predominantly Muslim countries), consume less alcohol or declare drinking less when seeking assistance. Additionally, it is possible, although extremely difficult to prove, that they may not seek assistance if they have been assaulted after consuming alcohol.

Considering foreign women, in our research, the nationalities predominantly represented were Romania, Morocco, Peru, and Nigeria. When comparing the number of cases with the official population of each nationality residing in Turin, it is evident that the ratio of victims to the total number of women from those nationalities is significantly higher for these four nationalities (especially Peru and Nigeria) compared to Italian women. Within these populations, the proportion of Peruvian women who reported alcohol consumption (0.329) is notably higher compared to Nigerian women (0.116). Conversely, the latter have a much higher proportion (0.580) of cases where alcohol consumption was not reported, indicating a contrasting pattern between the two groups. This difference between the two nationalities can find an explanation in the stories of women reported in their medical records. Often, Peruvian women stated that they consumed alcohol during parties/gatherings with compatriots, where they were later abused. The majority of Nigerian women who sought help at the SVS Centre, on the other hand, told stories of abuse suffered while engaging in prostitution. These observations emphasise the importance of considering both the nationality and type of alcohol consumption when analysing the prevalence of assault among foreign women. It is crucial to identify the underlying factors contributing to these differences and to develop targeted interventions that address the specific needs and vulnerabilities of each population.

Our analysis also sheds light on the relationship between alcohol consumption and the location where violence was perpetrated. It is interesting to note that, in public places and in someone else’s home, the prevalence of violence was higher among women who had consumed alcohol compared to the control group. This suggests a potential association between alcohol consumption and an increased vulnerability to violence in these specific settings. On the other hand, the control group showed a significantly higher occurrence of violence in one’s own home. Alcohol consumption influences the location of violence and the identity of assailants. Indeed, women raped in their own homes and someone else’s home showed different profiles based on alcohol consumption. Italian women who consumed alcohol were more frequently victimised in cases of domestic violence, while foreign women were more prevalent in the control group. Age also played a role, with women aged 18–25 facing higher risks in public places after alcohol consumption. Non-drinking women were more often victimised in their own homes. These findings emphasize the need for targeted prevention strategies considering specific factors in sexual assault cases.

When considering the total population of our study, the assailant was unknown in 36.3% of cases, while 57.8% of victims knew their aggressor; these findings are comparable with those reported by Loder and Robinson [44] in their retrospective US survey, while there was a reversal from the study of Ricard-Gauthier [33].

The aggressor is the partner significantly more frequently in the control group, and particularly in foreign women, suggesting that he is responsible for violence, especially in the absence of alcohol consumption. Moreover, the aggressor is an acquaintance in most of the minor cases, particularly if they consumed alcohol.

The majority of women who consumed alcohol accessed the SVS within the first 24 h after the event. We can speculate that this difference is due to the presence of other symptoms pushing the search for medical assistance. In fact, at clinical presentation, women who consumed alcohol had a higher rate of lesions, namely, multiple injuries excluding the genital area and neurological symptoms (mostly amnesia and confusion).

Women who experienced violence after consuming alcohol declared significantly higher consumption rates for benzodiazepines, Z-drugs, and antidepressants. In the context of chronic treatment, it is noteworthy that women who experienced SV after consuming alcohol had higher utilisation of benzodiazepines and antidepressants. Consistently, this increased usage and the association between benzodiazepine consumption and unhealthy alcohol use was suggested in Hirschtritt’s [45] study. When benzodiazepines and alcohol are used concurrently, several toxicological interactions with significant clinical implications can occur [46].

While there was no difference between the alcohol and control groups in the rate of women consuming SNC-active medications, the difference was significant when considering recreational drugs, whose use was higher within the alcohol group. Moreover, the victims in the control group who declared their consumption mostly affirmed that they were forced to use these drugs.

We also performed adjusted assessments of factors related to sexual aggression, considering alcohol intake and the location of the violence as outcome variables. The multiple regression analysis revealed that Italian women and those aged 18–25 and 26–35 years had higher odds of being injured after consuming alcohol. Violence by unknown individuals or acquaintances and in someone else’s private home was more likely when alcohol was involved.

Overall, this research sheds light on the interplay between alcohol, specific demographics, and the context of sexual violence, providing, consistent with the reliability of a retrospective study, valuable insights into the factors associated with the risk of injury in a population living in the northwestern Italian area.

Despite the large population examined, our results should be interpreted in the context of several limitations. The analysis stopped in 2019, because with the advent of the COVID-19 pandemic, people’s habits and societal conditions changed, including the levels and patterns of alcohol consumption and violence rates [47,48,49,50]. Moreover, the information on alcohol/drug consumption was based on declarations from the women. One limit common to retrospective studies is that their reliability is contingent on the accuracy and completeness of existing records, with incomplete or inaccurate data posing a threat to the overall study quality. Additionally, we enrolled only one-third of the total control population, although this was randomly chosen in order to avoid any selection bias. Finally, it is difficult to compare our results with analogous studies since, to the best of our knowledge, few published surveys had a case-control design.

## 5. Conclusions

The study conducted an explorative retrospective evaluation of sexual violence episodes involving women who had consumed alcohol before being assaulted. Our findings show that alcohol consumption is associated with specific risk factors and injury patterns. The study highlights the need for targeted prevention strategies when addressing the complex interplay of alcohol demographics and sexual violence. Our findings can guide targeted interventions and prevention strategies to address sexual violence and inform communities facing similar challenges.

## Figures and Tables

**Table 1:** The table shows the number of women in each group who reported taking medication, by category. The total is higher than the number of women who declared drug intake because some of them were taking more than one drug.

	Alcohol Group	Control Group
Antidepressant	15 *	6
Antiepileptic	8	8
Antipsychotic	13	10
Benzodiazepines and Z-drugs	29 **	21
Medications for addiction treatment	4	1
Other	1	1

* *p* = 0.016 vs. control; ** *p* = 0.008 vs. control.

**Table 2:** Multiple logistic regression model, considering sexual aggression related to alcohol consumption (yes vs. no) as the outcome variable.

	Adjusted Effect		
	OR	95% CI	*p*-Value
**Nationality**			
Foreign	1.00		
Italian	1.72	(1.15, 2.56)	0.008
**Age (years)**			
14–17	1.07	(0.57, 2.00)	0.834
18–25	2.80	(1.65, 4.76)	<0.001
26–35	2.03	(1.12, 3.67)	0.019
>35	1.00		
**Location of aggression**			
Other	1.00		
Public place	1.29	(0.77, 2.17)	0.335
Own home	0.88	(0.45, 1.72)	0.699
Someone else’s home	2.15	(1.23, 3.75)	0.007
**Assailant**			
Partner	1.00		
Unknown	5.25	(2.35, 11.7)	<0.001
Acquaintance	4.51	(2.06, 9.87)	<0.001

Bold defines the categories considered in the multiple logistic regression.

**Table 3:** Multiple logistic regression model, considering the location of sexual violence (own private home vs. other) as the outcome variable.

	Adjusted Effect		
	OR	95% CI	*p*-Value
**Nationality**			
Italian	1.00		
Foreign	1.75	(1.7, 2.88)	0.026
**Age (years)**			
14–17	1.00		
18–25	1.37	(0.63, 2.98)	0.424
26–35	1.94	(0.86, 4.38)	0.113
>35	3.16	(1.45, 6.89)	0.004
**Assailant**			
Unknown	1.00		
Partner	12.76	(6.47, 25.17)	<0.001
Acquaintance	2.80	(1.53, 5.14)	0.001
**Intake of alcohol**			
Yes	1.00		
No	1.72	(1.01, 2.97)	0.05

Bold defines the categories considered in the multiple logistic regression.

## Data Availability

The data presented in this study are available on request from the corresponding author. The data are not publicly available due to the topics and data reported in this article are extremely delicate and sensitive, given that they refer to episodes of violence suffered by women located in a well-localized geographical area.

## References

[1] World Health Organization Violence against Women. https://www.who.int/news-room/fact-sheets/detail/violence-against-women#:~:text=Estimates%20published%20by%20WHO%20indicate,sexual%20violence%20in%20their%20lifetime.

[2] Center for Disease Control and Prevention Risk and Protective Factors. https://www.cdc.gov/violenceprevention/sexualviolence/riskprotectivefactors.html.

[3] Vos T., Astbury J., Piers L.S., Magnus A., Heenan M., Stanley L., Walker L., Webster K. (2006). Measuring the impact of intimate partner violence on the health of women in Victoria, Australia. Bull. World Health Organ..

[4] Monk L., Jones A. (2014). Alcohol consumption as a risk factor for sexual assault: A retrospective analysis. J. Forensic Leg. Med..

[5] Norona J.C., Borsari B., Oesterle D.W., Orchowski L.M. (2021). Alcohol Use and Risk Factors for Sexual Aggression: Differences According to Relationship Status. J. Interpers. Violence.

[6] Abbey A., Wegner R., Woerner J., Pegram S.E., Pierce J. (2014). Review of survey and experimental research that examines the relationship between alcohol consumption and men’s sexual aggression perpetration. Trauma Violence Abus..

[7] Lippy C., DeGue S. (2016). Exploring Alcohol Policy Approaches to Prevent Sexual Violence Perpetration. Trauma Violence Abus..

[8] Kingree J.B., Thompson M. (2015). A comparison of risk factors for alcohol-involved and alcohol-uninvolved sexual aggression perpetration. J. Interpers. Violence.

[9] Boles S.M., Miotto K. (2003). Substanceabuse and violence: A review of the literature. Aggress. Violent Behav..

[10] Abbey A., Zawacki T., Buck P.O., Clinton A.M., McAuslan P. (2004). Sexual assault and alcohol consumption: What do we know about their relationship and what types of research are still needed?. Aggress. Violent Behav..

[11] Abbey A. (2011). Alcohol’s role in sexual violence perpetration: Theoretical explanations, existing evidence and future directions. Drug Alcohol Rev..

[12] Room R., Babor T., Rehm J. (2005). Alcohol and public health. Lancet.

[13] Devries K.M., Child J.C., Bacchus L.J., Mak J., Falder G., Graham K., Watts C., Heise L. (2014). Intimate partner violence victimization and alcohol consumption in women: A systematic review and meta-analysis. Addiction.

[14] George W.H., Stoner S.A. (2000). Understanding acute alcohol effects on sexual behavior. Annu. Rev. Sex Res..

[15] George W.H. (2019). Alcohol and Sexual Health Behavior: “What We Know and How We Know It”. J. Sex Res..

[16] Tedor M.F., Quinn L.M., Wilsnack S.C., Wilsnack R.W., Greenfield T.K. (2018). Gender and Country Differences in Alcohol-Aggression Expectancy and Alcohol-Related Intimate Partner Violence. Deviant Behav..

[17] Busardò F.P., Varì M.R., di Trana A., Malaca S., Carlier J., di Luca N.M. (2019). Drug-facilitated sexual assaults (DFSA): A serious underestimated issue. Eur. Rev. Med. Pharmacol. Sci..

[18] Costa Y.R.S., Lavorato S.N., Baldin J. (2020). Violence against women and drug-facilitated sexual assault (DFSA): A review of the main drugs. J. Forensic Leg. Med..

[19] Fiorentin T.R., Logan B.K. (2019). Toxicological findings in 1000 cases of suspected drug facilitated sexual assault in the United States. J. Forensic Leg. Med..

[20] Scott-Ham M., Burton F.C. (2005). Toxicological findings in cases of alleged drug-facilitated sexual assault in the United Kingdom over a 3-year period. J. Clin. Forensic Med..

[21] ElSohly M.A., Salamone S.J. (1999). Prevalence of drugs used in cases of alleged sexual assault. J. Anal. Toxicol..

[22] Hagemann C.T., Helland A., Spigset O., Espnes K.A., Ormstad K., Schei B. (2013). Ethanol and drug findings in women consulting a Sexual Assault Center--associations with clinical characteristics and suspicions of drug-facilitated sexual assault. J. Forensic Leg. Med..

[23] Johansen S.S. (2017). Detection of the antipsychotic drug quetiapine in the blood, urine and hair samples of the victim of a drug-facilitated sexual assault. Forensic Sci. Int..

[24] Melchior S.E., Nielsen M.K.K., Oropeza A.R., Banner J., Johansen S.S. (2023). Detection of scopolamine in urine and hair in a drug-facilitated sexual assault. Forensic Sci. Int..

[25] Mognetti B., Bo M., Berta G.N., Canavese A., Castagna P., Collini F., Santa V., Salomone A., Gino S. (2022). Sexual Harassments Related to Alcohol and Drugs Intake: The Experience of the Rape Centre of Turin. Int. J. Environ. Res. Public Health.

[26] Tuttitalia.it Cittadini Stranieri 2021. https://www.tuttitalia.it/piemonte/provincia-di-torino/statistiche/cittadini-stranieri-2021/.

[27] Grant B.F., Chou S.P., Saha T.D., Pickering R.P., Kerridge B.T., Ruan W.J., Huang B., Jung J., Zhang H., Fan A. (2017). Prevalence of 12-Month Alcohol Use, High-Risk Drinking, and DSM-IV Alcohol Use Disorder in the United States, 2001–2002 to 2012–2013: Results from the National Epidemiologic Survey on Alcohol and Related Conditions. JAMA Psychiatry.

[28] RAPPORTO ISTISAN 2022. Epidemiologia e Monitoraggio Alcol-Correlato in Italia e Nelle Regioni. https://www.epicentro.iss.it/alcol/pdf/RAPPORTO%20ISTISAN%20ALCOL%202022%20Osservatorio%20Nazionale%20Alcol.pdf.

[29] Bloomfield K., Gmel G., Neve R., Mustonen H. (2001). Investigating Gender Convergence in Alcohol Consumption in Finland, Germany, The Netherlands, and Switzerland: A Repeated Survey Analysis. Subst. Abus..

[30] Pathak H., Frieling H., Bleich S., Glahn A., Heberlein A., Haschemi Nassab M., Hillemacher T., Burkert A., Rhein M. (2017). Promoter Polymorphism rs886205 Genotype Interacts with DNA Methylation of the ALDH2 Regulatory Region in Alcohol Dependence. Alcohol Alcohol..

[31] Förstera B., Castro P.A., Moraga-Cid G., Aguayo L.G. (2016). Potentiation of Gamma Aminobutyric Acid Receptors (GABAAR) by Ethanol: How Are Inhibitory Receptors Affected?. Front. Cell. Neurosci..

[32] Li L., Shen X., Zeng G., Huang H., Chen Z., Yang J., Wang X., Jiang M., Yang S., Zhang Q. (2023). Sexual violence against women remains problematic and highly prevalent around the world. BMC Womens Health.

[33] Ricard-Gauthier D., Abdulcadir J., Tony F., Yaron M. (2021). Care of women and girls after sexual assault in Geneva: A descriptive study between 2005 and 2014. Eur. J. Obstet. Gynecol. Reprod. Biol..

[34] Mizrachi Y., Bar J., Barda G. (2020). Characteristics and trends of sexual assaults in Israel—A large cohort study of 3941 victims. Acta Obstet. Gynecol. Scand..

[35] Morgan L., Long L. (2018). Female perpetrated sexual offences reported to a London sexual assault referral centre. J. Forensic Leg. Med..

[36] Vogt E.L., Jiang C., Jenkins Q., Millette M.J., Caldwell M.T., Mehari K.S., Marsh E.E. (2022). Trends in US Emergency Department Use After Sexual Assault, 2006–2019. JAMA Netw. Open.

[37] Abbey A., Zawacki T., Buck P.O., Clinton A.M., McAuslan P. (2001). Alcohol and sexual assault. Alcohol Res. Health.

[38] Choi K.W., Na E.J., Hong J.P., Cho M.J., Fava M., Mischoulon D., Cho H., Jeon H.J. (2018). Alcohol-induced disinhibition is associated with impulsivity, depression, and suicide attempt: A nationwide community sample of Korean adults. J. Affect. Disord..

[39] Kanin E.J. (1985). Date rapists: Differential sexual socialization and relative deprivation. Arch. Sex. Behav..

[40] Mosher D.I., Anderson R.D. (1986). Macho personality, sexual aggression, and reactions to guided imagery of realistic rape. J. Res. Pers..

[41] Graham K., Bernards S., Wayne Osgood D., Abbey A., Parks M., Flynn A., Dumas T., Wells S. (2014). “Blurred lines?” Sexual aggression and barroom culture. Alcohol. Clin. Exp. Res..

[42] NIAAA Underage Drinking. https://www.niaaa.nih.gov/publications/brochures-and-fact-sheets/underage-drinking.

[43] Sánchez-Puertas R., Vaca-Gallegos S., López-Núñez C., Ruisoto P. (2022). Prevention of Alcohol Consumption Programs for Children and Youth: A Narrative and Critical Review of Recent Publications. Front. Psychol..

[44] Loder R.T., Robinson T.P. (2020). The demographics of patients presenting for sexual assault to US emergency departments. J. Forensic Leg. Med..

[45] Hirschtritt M.E., Palzes V.A., Kline-Simon A.H., Kroenke K., Campbell C.I., Sterling S.A. (2019). Benzodiazepine and unhealthy alcohol use among adult outpatients. Am. J. Manag. Care.

[46] Tanaka E. (2002). Toxicological interactions between alcohol and benzodiazepines. J. Toxicol. Clin. Toxicol..

[47] Rehm J., Kilian C., Ferreira-Borges C., Jernigan D., Monteiro M., Parry C.D.H., Sanchez Z.M., Manthey J. (2020). Alcohol use in times of the COVID 19: Implications for monitoring and policy. Drug Alcohol. Rev..

[48] Sohi I., Chrystoja B.R., Rehm J., Wells S., Monteiro M., Ali S., Shield K.D. (2022). Changes in alcohol use during the COVID-19 pandemic and previous pandemics: A systematic review. Alcohol. Clin. Exp. Res..

[49] Al Mamun F., Hosen I., Mamun M.A. (2021). Sexual violence and rapes’ increment during the COVID-19 pandemic in Bangladesh. EClinicalMedicine.

[50] Muldoon K.A., Denize K.M., Talarico R., Fell D.B., Sobiesiak A., Heimerl M., Sampsel K. (2021). COVID-19 pandemic and violence: Rising risks and decreasing urgent care-seeking for sexual assault and domestic violence survivors. BMC Med..

